# Association Between Human Gut Microbiome and N-Glycan Composition of Total Plasma Proteome

**DOI:** 10.3389/fmicb.2022.811922

**Published:** 2022-04-29

**Authors:** Vyacheslav A. Petrov, Sodbo Zh. Sharapov, Lev Shagam, Arina V. Nostaeva, Marija Pezer, Dalin Li, Maja Hanić, Dermot McGovern, Edouard Louis, Souad Rahmouni, Gordan Lauc, Michel Georges, Yurii S. Aulchenko

**Affiliations:** ^1^Unit of Animal Genomics, Grappe Interdisciplinaire de Génoprotéomique Appliquée-Institute, University of Liège, Liège, Belgium; ^2^Central Research Laboratory, Ministry of Healthcare of Russian Federation, Federal State Budget Educational Institution of Higher Education, Siberian State Medical University, Tomsk, Russia; ^3^Laboratory of Glycogenomics, Institute of Cytology and Genetics, Novosibirsk, Russia; ^4^Laboratory of Theoretical and Applied Functional Genomics, Novosibirsk State University, Novosibirsk, Russia; ^5^Genos Ltd., Zagreb, Croatia; ^6^Cedars-Sinai Medical Center, The F. Widjaja Foundation Inflammatory Bowel and Immunobiology Research Institute, Los Angeles, CA, United States; ^7^Glycoscience Research Laboratory, Genos Ltd., Zagreb, Croatia; ^8^Laboratory of Translational Gastroenterology, Grappe Interdisciplinaire de Génoprotéomique Appliquée-Institute, University of Liège, Liège, Belgium; ^9^Department of Biochemistry and Molecular Biology, Faculty of Pharmacy and Biochemistry, University of Zagreb, Zagreb, Croatia

**Keywords:** mucosal microbiome, plasma N-glycome, 16S sequencing, IgG N-glycome, *Bilophila*

## Abstract

Being one of the most dynamic entities in the human body, glycosylation of proteins fine-tunes the activity of the organismal machinery, including the immune system, and mediates the interaction with the human microbial consortium, typically represented by the gut microbiome. Using data from 194 healthy individuals, we conducted an associational study to uncover potential relations between the gut microbiome and the blood plasma N-glycome, including N-glycome of immunoglobulin G. While lacking strong linkages on the multivariate level, we were able to identify associations between alpha and beta microbiome diversity and the blood plasma N-glycome profile. Moreover, for two bacterial genera, namely, *Bilophila* and *Clostridium innocuum*, significant associations with specific glycans were also shown. The study’s results suggest a non-trivial, possibly weak link between the total plasma N-glycome and the gut microbiome, predominantly involving glycans related to the immune system proteins, including immunoglobulin G. Further studies of glycans linked to microbiome-related proteins in well-selected patient groups are required to conclusively establish specific associations.

## Introduction

Protein glycosylation is a posttranslational modification that consists of the binding of carbohydrate chains, or glycans, to the polypeptide backbone. Such modifications regulate protein activity and their half-life and even serve as a form of cellular memory, reflecting the past and current processes in a cell, in both physiological and pathological conditions (Lauc et al., 2016). Changes in the plasma glycome profile are evident for a variety of diseases, including congenital and multifactorial disorders (Dotz and Wuhrer, 2019). By affecting the activity of immunoglobulins and immune receptors (Wolfert and Boons, 2013; Cambay et al., 2020), glycosylation potentially exerts its influence on the interaction between the host organism and its microbiome. Kudelka et al. (2020) showed that the gut microbial community can itself manipulate the glycosylation profile of the enteral epithelium, co-regulating the gut homeostasis along with the host, but whether these effects remain local or extend across organisms is unknown. The present study aims to identify, for the first time, the potential links between the total plasma N-glycome profile and the gut mucosal microbiome composition. For this, we performed an analysis of the association between the gut microbiome and the relative abundance of different glycans attached to blood plasma proteins (including immunoglobulin G) in a group of individuals from the Correlated Expression and Disease Association Research (CEDAR) cohort consisting a total of 323 well-characterized healthy individuals with intestinal biopsies (ileum, transverse colon, and rectum) available (Momozawa et al., 2018).

## Materials and Methods

### Studied Population

The analyzed population samples included 194 healthy Europeans visiting the Academic Hospital of the University of Liège as part of a national screening campaign for colon cancer. The enrolled individuals were not suffering from any autoimmune or inflammatory disease and were not taking corticosteroids or non-steroid anti-inflammatory drugs, with the exception of low doses of aspirin to prevent thrombosis (Momozawa et al., 2018).

### 16S rRNA Gene Sequencing

DNA was extracted from intestinal biopsies of the ileum, the transverse colon, and the rectum using the QIAamp DNA Stool Mini Kit (QIAgen, Germany). Three fragments of the 16S rRNA gene with variable regions, namely, V1–V2, V3–V4, and V5–V6, were amplified independently. (Primer sequences are listed in Supplementary Table 4). For library preparation, locus-specific deep sequencing was performed using a protocol of two PCR strategies (Jervis-Bardy et al., 2015). The paired-end libraries were sequenced on the Illumina MiSeq instrument with a read length of 2 × 300 bp.

### Microbiome Data Processing

The read lengths with QV 20 were trimmed from the 3′ end and demultiplexed, the primer sequences were removed, and then, reads mapping to the human genome were removed using the BBTools suite (Bushnell, 2014). The pipeline was constructed using Snakemake (Köster and Rahmann, 2012). A further analysis was performed by QIIME 2 2018.11 (Bolyen et al., 2019). As a result, 180.5 mln paired-end reads were obtained, of which 156.8 mn reads were retained after quality filtering. The paired-end reads were denoised and clustered by the DADA2 plugin (Callahan et al., 2016) using batch-specific trimming length parameters yielding 9.1 ± 2.0 K amplicon sequence variants (ASVs) per run for V1V2, 4.5 ± 1.6 K for V3V4, and 6.8 ± 0.67 K for V5V6 amplicon. Taxonomy was assigned at a genus level to all ASVs using the q2-feature-classifier (Bokulich et al., 2018) classify−sklearn naïve Bayes taxonomy classifier against the SILVA ribosomal RNA database release 132 (Quast et al., 2013). Accordingly, we obtained three microbiota profiles for each of the intestinal locations.

A further analysis was performed in the R language, version 3.6.1 (R Core Team, 2019). Given the fact that the contamination from reagents can significantly distort the observed taxa-abundance distributions as described elsewhere (Salter et al., 2014; de Goffau et al., 2018; Eisenhofer et al., 2019), we aimed to identify taxa that demonstrate abnormal behavior characteristics for contaminants. The list of taxa determined in negative controls is given in Supplementary Table 5. We modeled the taxa-abundance distribution to reveal genera that behave as contaminants taking advantages of (i) the presence of biological replicates for 25 sample–location combinations, (ii) the dependence of taxon abundance on the sample coverage depth for some taxa, and (iii) the batch effects traceable due to the presence of nine sequencing batches. For centered log-ratio-transformed data (zero read counts were imputed by a minimal fraction of the taxon across all samples and locations), we revealed genera that matched either of the conditions: (i) a significant (*p* < 0.05 after Benjamini–Hochberg correction) negative correlation with the coverage depth, (ii) low consistency across biological replicates (Spearman’s correlation *r* < 0.3), (iii) relatively low consistency across biological replicates (*r* < 0.4) and not being characteristic for human gut microbiota, and (iv) significant run discordance (*p* < 0.05 after the Benjamini–Hochberg correction) and not being characteristic for human gut microbiota. Run discordance and correlation with the coverage depth were calculated using the ANOVA of a linear model with the following explanatory variables: patients’ age, sex, BMI, smoking status, sample collection batch, intestinal location, and sequencer run batch crossed with 16S rRNA amplicon nested into location. On average, across locations and amplicons, 2.5% of sequencing reads were mapped to contaminant taxa revealed above, which were removed from further analysis.

Only the samples with at least 10,000x (for V1–V2 and V5–V6) or 5,000x (for V3–V4) coverage were subjected to further analysis. Taxa with < 0.01% average abundance in any location–amplicon combination were removed. For other taxa, zero read counts were imputed by a minimal fraction of the taxon across all samples and locations. After performing a centered log-ratio (CLR) transformation, the data were corrected for technical batch effects (sequencing batch effect, amplicon, and location) using a linear mixed model implemented in the lme4 package (Bates et al., 2015):

taxon abundance ∼ (Run:Amplicon)%in%Location + (1| Date.collection) + Location + Amplicon.

Then, nine available taxa-abundance distributions per sample were averaged to get one more precise measurement for each individual. Patients’ age, sex, body mass index, and smoking status were considered as possible covariates. To additionally refine the data, we performed PCA using the ade4 package (Bougeard and Dray, 2018) and added the values of the first four principal components (explaining 24.2% of the total variance) to the covariates list.

### Plasma N-Glycome Quantification

Plasma N-glycome quantification of the CEDAR samples was performed at Genos^1^ by applying the following protocol. Plasma N-glycans were enzymatically released from proteins by incubation with PNGase F, fluorescently labeled with 2-aminobenzamide, and cleaned up from the excess of reagents by hydrophilic interaction liquid chromatography–solid-phase extraction (HILIC–SPE), as previously described by Akmačić et al. (2015). The fluorescently labeled and purified N-glycans were separated by HILIC on a Waters BEH Glycan chromatography column, 150 × 2.1 mm, 1.7 μm BEH particles, installed on an Acquity UPLC instrument (Waters, Milford, MA, United States) consisting of a quaternary solvent manager, a sample manager, and a fluorescence detector set with excitation and emission wavelengths of 250 and 428 nm, respectively. Following chromatography conditions previously described in detail (Akmačić et al., 2015), total plasma N-glycans were separated into 39 peaks. The amount of N-glycans in each chromatographic peak was expressed as a percentage of the total integrated area. Glycan peaks (GPs), quantitative measurements of glycan levels, were defined by the automatic integration of intensity peaks on a chromatogram. The composition of the major N-glycan structures in chromatographic peaks had been assigned previously (Zaytseva et al., 2020).

### Immunoglobulin G N-Glycome Quantification

IgG was isolated from 10 μl of human plasma per sample using a 96-well CIM Protein G monolithic plate (BIA Separations, Ajdovščina, Slovenia). Subsequently, IgG N-glycans were enzymatically released by incubation with PNGase F, fluorescently labeled with 2-aminobenzamide, and cleaned up by HILIC–SPE as previously described (Trbojević-Akmačić et al., 2017). Following previously established chromatographic parameters, the fluorescently labeled and purified IgG N-glycans were separated into 24 glycan peaks by HILIC on a Waters BEH Glycan chromatography column, 100 × 2.1 mm, 1.7 μm BEH particles, installed on an Acquity UPLC instrument (Waters, Milford, MA, United States) (Trbojević-Akmačić et al., 2017). The amount of N-glycans in each chromatographic peak was expressed as a percentage of the total integrated area, and their N-glycan composition had been assigned previously (Pučić et al., 2011).

### Harmonization of Glycan Peaks

The similarity of the order of the glycan peaks (GPs) on a UPLC chromatogram among studies is known (Sharapov et al., 2019). However, depending on the cohort, some peaks located near one another might be indistinguishable. In order to make the protocol of our study applicable to other cohorts and promote replication studies, we performed harmonization of the total plasma N-glycome samples using a recently developed protocol (Sharapov et al., 2019). In brief, according to the major glycostructures within the GPs, we manually created the table of correspondence between different GPs (or sets of GPs) àcross several cohorts, where plasma glycome was measured using the UPLC technology. Then, based on this table of correspondence, we defined the list of 36 harmonized GPs (listed in Supplementary Table 6) and the harmonization algorithm for each cohort, including CEDAR. Using this algorithm, the total plasma N-glycome profile of each CEDAR sample was harmonized into 36 GPs.

### Normalization, Batch Correction of Glycan Peaks, and Derived Trait Calculation

Normalization and batch correction were performed on the harmonized UPLC glycan data. We used the total area normalization (the area of each GP was divided by the total area of the corresponding chromatogram). From the 36 directly measured glycan traits, 81 derived traits were calculated (Supplementary Table 6). These derived traits average glycosylation features such as branching, galactosylation, and sialylation across different individual glycan structures, and consequently, they may be more closely related to individual enzymatic activity. For the original traits, CLR transformation from the “compositions” R package (van den Boogaart and Tolosana-Delgado, 2008) was implemented to account for the compositional nature of the data (Galligan et al., 2013). For the derived traits, different approaches of compositional transformations were used depending on the type of the features (Supplementary Table 6). In brief, if a derived trait represented a relative concentration of the sum of some original traits (e.g., the sum of PGP1, PGP2, and PGP3 in all 117 traits) in the whole composition, then the derived trait was computed as the sum of these original traits followed by CLR transformation [CLR(sum(PGP1.PGP3), other traits)]. If a derived trait represented the sum of original traits in some repertoire of glycans (e.g., the sum of PGP1, PGP2, and PGP3 in the first 10 traits), then at the first stage, the subcomposition of this repertoire was obtained [PGP1.PGP10/sum(PGP1.PGP10)] and the second stage is similar to the previous case. Finally, if a derived trait represented the ratio between two parts of the composition, the isometric log-ratio transformation was used (Greenacre, 2018).

### Polygenic Score Derivation and Mendelian Randomization

A polygenic risk score (PRS) aggregates the effects of many genetic variants into a single number, which predicts genetic predisposition for the phenotype. In the standard approach, the PRS is a linear combination of linear regression effect size estimates and allele counts at single-nucleotide polymorphisms (SNPs).

We developed PRS models using the SBayesR (Lloyd-Jones et al., 2019) method that utilized summary statistics from a genome-wide association study (GWAS). This tool reweighs the effect of each variant according to the marginal estimate of its effect size, statistical strength of association, the degree of correlation between the variant and other variants nearby, and tuning parameters. Also, the SBayesR method requires a GCTB (Lloyd-Jones et al., 2019)—a compatible LD matrix file computed using individual-level data from a reference population. For these analyses, we used publicly available shrunk sparse GCTB LD matrix computed from a random set of 50,000 individuals of European ancestry from the UK Biobank dataset (Bycroft et al., 2018; Lloyd-Jones et al., 2019). The models were validated in the CEDAR dataset, which was not part of the samples used for GWAS. The prediction accuracy was defined as the proportion of the variance of a phenotype that is explained by PRS values (R2). To calculate PRS based on the PRS model, we used PLINK2 software (Chang et al., 2015), where PRS values were calculated as a weighted sum of allele counts.

Associations between PRS values, acting as an instrumental variable, and the microbial genera abundance were checked in a linear regression analysis (Richardson et al., 2019).

### Statistical Analysis

Statistical analysis was conducted in R language, version 3.6.1 (R Core Team, 2019). The principal component analysis of the glycome data was performed using the standard prcomp function of stats R package. The associations were examined in a linear regression model. We separately tested associations between (i) the total plasma N-glycome and the gut microbiome composition; (ii) beta diversity and the total plasma N-glycome; alpha diversity and the total plasma N-glycome—both; (iii) the glycan traits; and (iv) the first 10 microbial principal components. Patients’ age, sex, body mass index (BMI), and smoking status were used as covariates. For the first model, the values of the first four microbial principal components were used as additional covariates. Before regression modeling, the bacterial abundances were quantile-normalized *via* qqnorm R function.

*P*-values were adjusted to multiple hypothesis testing with the Sidak correction procedure. Taking into consideration the possible correlations between hypotheses, the number of effective tests for Sidak correction was computed for both the glycome and microbiome data. For the estimation of the number of effective tests, the approach of Galwey (2009) implemented in the poolR package (Cinar and Viechtbauer, 2020) was used. Visualization was performed with the ggplot2 package (Wickham, 2009).

## Results

To access the gut mucosal microbiome composition, biopsies were collected from consented donors who visited the Department of Gastroenterology of Liege University Hospital in the framework of the Belgian colon cancer prevention program. Biopsies were collected from three different locations of the gut, namely, the ileum, the transverse colon, and the rectum. The study participants were selected based on their health records. The exclusion criteria included autoimmune diseases and any type of inflammatory bowel diseases, cancer or polyps found during colonoscopy, antibiotics and anti-inflammatory uptake at least 3 weeks prior to the biopsies collection, and absence of diarrhea. Biopsies were snap-frozen and kept at −80°C until DNA extraction. The three amplicons, namely, V1–V2, V3–V4, and V5–V6, were used to amplify microbial 16S rRNA genes. In total, nine Illumina MiSeq runs (three amplicons × three gut locations) were performed on 2012 samples collected from 336 patients and 40 negative controls for sequencing. DADA2 amplicon sequence variants were analyzed by the q2−feature−classifier trained on the Silva database to assign taxonomy at the genus level. Furthermore, we measured the total plasma N-glycome for 234 CEDAR samples and 15 standard samples, of which 230 samples passed quality control. Chromatograms for each sample were separated into 39 peaks and harmonized into 36 glycome peaks for easier comparison with other published research. In addition, based on shared structural features, 81 derived traits were calculated. Hereafter, we used “PGP_number” not only to refer to the originally measured and derived glycan traits but also to provide a description of the glycan structures along with their Oxford notation (Harvey et al., 2009).

Metagenomic and glycomic data were simultaneously available for 194 individuals (Table 1), thus allowing us to investigate the inter-omics relationships on different levels of detalization, from diversity and multivariate associations to individual linkages.

**TABLE 1:** Demographic information of the cohort studied.

Characteristic	Overall
Sample size	194
Age, mean (SD)	55.66 (13.05)
Body mass index, mean (SD)	26.37 (4.64)
**Ethnicity, absolute n (%)**	
Caucasian	159 (82.0)
Mediterranean	23 (11.9)
Mixed	12 (6.2)
Sex (males), absolute n (%)	82 (42.3)
Smoking status (smokers), absolute n (%)	45 (23.2)

**FIGURE 1:**
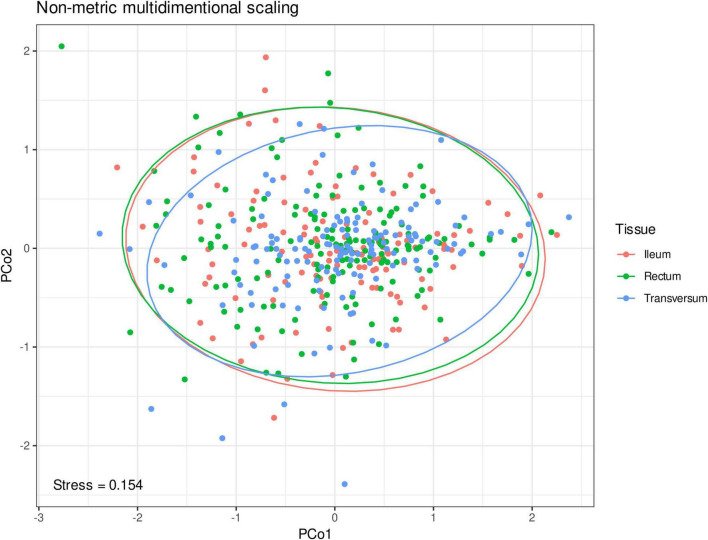
Non-metric multidimensional scaling of the microbial abundances on the genera level in Euclidean metric. The first two principal coordinates are shown. The color dots represent microbiome samples from the ileum (red), colon transversum (blue), and rectal mucosa (green).

The analysis was conducted on the level of genera. After removing the contaminants and low-abundance microorganisms, 145 microbial genera were retained and used for further analyses. Among them, *Bacteroides* [ileum 34.6 (standard deviation 17.5)%, transversum 33.7 (19.0)%, and rectum 31.6 (17.3)%], *Prevotella 9* [ileum 8.3 (13.0)%, transversum 9.9 (14.8)%, and rectum 8.6 (12.8)%], and *Faecalibacterium* [ileum 6.0 (3.5)%, transversum 5.0 (4.3)%, and rectum 5.4 (3.3)%] dominated in the microbiome of the studied individuals irrespective of their localization. According to the results of the permutational multivariate analysis of variance, interindividual variation explains beta diversity of the microbiome better than the bioptate localization (*p* = 0.0001, Figure 1), which motivates averaging of the microbiome to obtain a more precise measurement for each individual.

Univariate associations between levels of individual glycan traits and microbial genera were studied using a linear model. Before the regression analysis, the number of effective statistical tests for the total plasma N-glycome and the gut microbiome data was calculated. According to the effective statistical test estimation, there were 24 effective tests in the glycome data and 87 in the microbiome data, which give a product of 2,088 independent tests. Genera abundances were normalized and adjusted for technical batch effects, and the known covariates, such as patients’ age, sex, body mass index, and smoking status, and the first four microbial PCs were added to the model.

Microbiome alpha diversity was calculated with the Shannon index (Shannon, 1948). The regression analysis was performed to identify possible links between the plasma glycome profile and the gut microbiome diversity. Significant negative associations were found between alpha diversity and the percentage of sialylation of core-fucosylated galactosylated structures without bisecting GlcNAc [derived trait PGP37, FGS/(FG + FGS), *p* = 0.041] and the percentage of disialylation of core-fucosylated digalactosylated structures without bisecting GlcNAc [derived trait PGP43, FG2S2/(FG2 + FG2S1 + FG2S2), *p* = 0.044] (Table 2).

**TABLE 2:** Association between microbiome alpha diversity (Shannon index) and the plasma total N-glycome profile.

N-glycan trait	Regression beta coefficient	Beta standard error	Nominal *p*-value	Sidak-corrected *p*-value
PGP43 [FG2S2/(FG2 + FG2S1 + FG2S2)]	−1.213	0.385	0.0019*	0.0440
PGP37 [FGS/(FG + FGS)]	−1.270	0.400	0.0018*	0.0410
Glycomic principal component 5	0.275	0.096	0.0045^#^	0.0440

*Corrected for 24 tests (reflecting the effective number of glycomic traits). ^#^Corrected for 10 tests (the number of glycomic PCs tested).

We then computed the first 10 glycan PCs on 117 traits. An association between alpha diversity and the value of the fifth glycan principal component was identified (Table 2). This principal component had a positive correlation with glycan traits representing the abundances of FA2B [mostly linked to immunoglobulin G (Vučković et al., 2016) and A2G2 (mostly linked to serotransferrin Clerc et al., 2016; Supplementary Table 1] but a negative correlation with glycan traits representing the abundances of FA2BG2S2 (mostly attached to immunoglobulins M and A) (Clerc et al., 2016) and FA2G2S2 (attached to various N-glycoproteins, mostly secreted to the bloodstream by the liver) (Supplementary Table 1).

To check the interplay between microbial communities and the plasma glycome profile, the Mantel correlation and the Procrustes analysis with 9,999 permutations were used. The result did not support a strong interrelation between the studied omics (Mantel *R* = −0.014, *p* = 0.63; Procrustes correlation = 0.22, *p* = 0.16). However, the individual glycan traits associated with the microbiome of the studied individuals, namely, traits PGP43 and PGP37, were positively correlated with the microbiome-derived sixth principal component (Table 3 and Supplementary Table 2).

**TABLE 3:** Association between microbiome beta diversity (principal component 6) and the total plasma N-glycome profile.

N-glycan trait	Regression beta coefficient	Beta standard error	Nominal *p*-value	Sidak-corrected *p*-value*
PGP43 [FG2S2/(FG2 + FG2S1 + FG2S2)]	2.992	0.734	6.8E-05	0.0161
PGP37 [FGS/(FG + FGS)]	3.013	0.766	0.0001	0.0280

*The multiple testing correction was made accounting for 240 tests (24 × 10, where 24 is the effective number of glycomic traits and 10 is the number of microbiome PCs).

**FIGURE 2:**
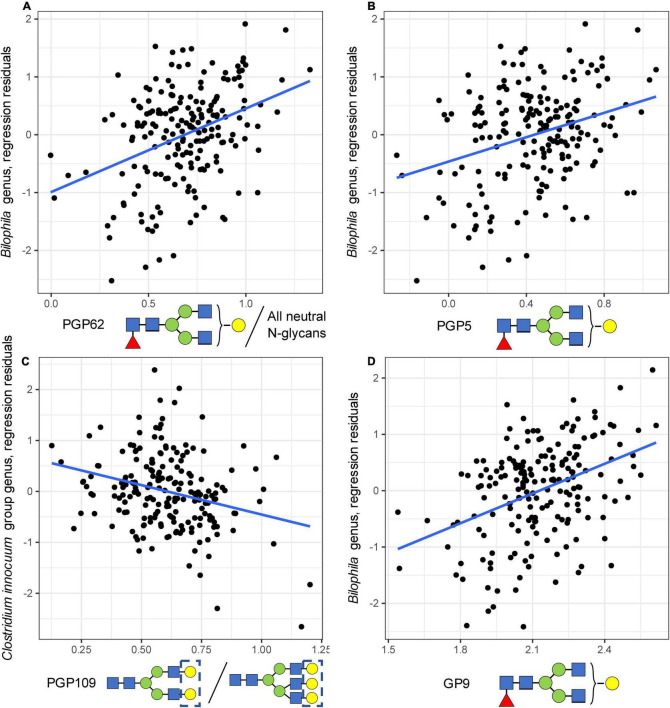
Univariate associations between microbial genera and glycan traits. On the plot, dots represent samples, and the regression line is shown in black. **(A)** The association between the abundance of *Bilophila* genus and the level of FA2[3]G1 in total neutral plasma N-glycans. **(B)** The association between the abundance of *Bilophila* genus and the level of FA2[3]G1 in total plasma N-glycans. **(C)** The association between the abundance of the *Clostridium innocuum* group genus and the ratio of disialylated and trisialylated trigalactosylated structures in total plasma N-glycans. **(D)** Technical validation of an association between IgG FA2[3]G1 glycan level and the abundance of *Bilophila* genus.

In the regression analysis, 981 bacterial-glycan pairs out of 16,965 pairs tested, including all glycan traits and 117 out of 145 bacterial genera, had a nominal *p*-value below the 0.05 threshold (Supplementary Table 3). This indicates an enrichment (*p*-value of binomial test = 0.0047) of the *p*-value distribution for significant *p*-values. Three bacterial–glycan pairs remained significant after adjustment for multiple testing. Specifically, we identified an association between the abundance of *Bilophila* genus and the level of FA2[3]G1 in total neutral plasma glycans [PGP62 trait, beta = 1.600 (0.278), nominal *p* = 4.24e-08, and Sidak-corrected *p* = 0.00009, Figure 2A], as well as the level of FA2[3]G1 in total plasma glycans [PGP5 trait, beta = 1.164 (0.246), nominal *p* = 4.44e-06, and Sidak-corrected *p* = 0.009, Figure 2B]. The abundance of the *Clostridium innocuum* group (an ASV defined on the genus level) demonstrated a negative association with the ratio of disialylated and trisialylated trigalactosylated structures in total plasma N-glycans [PGP109, G3S2/G3S3, beta = −1.460 (0.331), nominal *p* = 1.74e-05, and Sidak-corrected *p* = 0.036, Figure 2C].

In addition, the univariate association analysis was performed on levels of microbial phyla and families. We estimated the number of effective statistical tests as 11 at the phylum level and 69 at the family level, which, together with the genus level, resulted in 167 tests for microbiome data. The given 24 effective tests for the glycomic data provide an estimate of 4,008 independent tests in total. In this additional analysis, we did not identify significant associations on the phylum level. However, the abundance of the bacterial family, Tannerellaceae, was shown to be negatively associated with the levels of FA2[3]G1 in total plasma glycans, percentage of neutral glycan structures, and monogalactosylated structures in total plasma glycome (Supplementary Table 7 and Supplementary Figure 1). Identified genus-level associations remain significant after correction for additional statistical tests.

The validation of univariate findings on the genus level was performed in two steps. First, as N-glycosylation of immunoglobulin G (IgG) is the main source of FA2[3]G1 in the total plasma N-glycome (Clerc et al., 2016), we measured plasma IgG glycome profiles for 192 out of 194 individuals for the technical validation of association with FA2[3]G1. Using these data, we were able to validate the association between the abundance of FA2[3]G1 in IgG glycome and the abundance of *Bilophila* genus [beta = 1.899 (0.306), nominal *p* = 3.62e-09, Figure 2D].

As an external validation dataset, microbiome and total plasma N-glycome profiles from McHardy et al. (2013) were used. Given the differences in taxonomical databases used, metadata availability, and protocols of glycome and microbiome analysis between studies, it was possible to only study the association between the level of FA2[3]G1 in the total plasma N-glycome and the abundance of the *Bilophila*. The 47 samples for which microbiome and the total plasma N-glycome were available had an expected 56% power to replicate the results. We were unable to validate this association [beta = −109.192 (174.668), nominal *p* = 0.53], although the sign of association was consistent.

The fact that strong and robust genetic instruments are becoming available both for total plasma (Sharapov et al., 2019, 2021) and for IgG (Klarić et al., 2020) N-glycomic traits opens up an opportunity to investigate causal relations between plasma N-glycans and microbiome using Mendelian randomization. As instrumental variables for Mendelian randomization, we used polygenic scores computed for glycan traits that showed a significant association with the individual genera abundances. As a result, we found that the abundance of *Bilophila* genus was associated with a polygenic score for FA2[3]G1 in total plasma glycans [PGP5 trait, beta = 0.987 (0.429), nominal *p* = 0.0226] and suggestively associated with the polygenic score for FA2[3]G1 in total neutral plasma glycans [PGP5 trait, beta = 0.025 (0.137), nominal *p* = 0.0663]. This suggests a potentially causal link between the level of FA2[3]G1 and the abundance of *Bilophila* genus.

## Discussion

Overall, while our results suggest the presence of the association between the gut microbiota and the total plasma N-glycome, this interrelation seems to be relatively weak, with the largest proportion of variance explained to be equal to 14.7%. The strongest associations we showed were predominantly for N-glycans [FA2B, FA2(3)G1, and FA2BG2S2] linked to immunoglobulins. Both FA2G1 and *Bilophila* abundances showed a negative correlation with the risk of UC (Trbojević Akmačić et al., 2015; Hirano et al., 2018), which is consistent with the observed positive correlation between FA2[3]G1 glycan and *Bilophila*.

The *Clostridium innocuum* group showed an inverse association with the ratio of disialylated and trisialylated trigalactosylated structures in total plasma glycans. This ratio was reported to be negatively correlated with the blood level of C reactive protein, a known biomarker of inflammation (Suhre et al., 2019). *Clostridium innocuum*, the type species of the genus, treated as an unusual nosocomial agent, mainly caused infections in patients with immunodeficiency (Crum-Cianflone, 2009) and could be linked to antibiotic-associated diarrhea and may cause colitis (Chia et al., 2018).

In conclusion, in this study of 194 healthy individuals, we observed several associations between plasma N-glycome and the gut microbiome. We were able to perform technical validation of our strongest finding but were not able to replicate our finding in an independent dataset, perhaps due to its limited sample size (*n* = 47, expected power 56%). Taken together, this study’s results suggest the weak link between the gut microbiome and the composition of the total plasma N-glycome. The obtained results may suggest that a study of glycosylation of specific proteins, potentially connected with the microbiome, could be a more fruitful approach than an untargeted analysis performed here. One could also consider taking into account additional covariates, such as blood groups status, that may influence both the microbiome and the glycome.

## Data Availability Statement

The datasets presented in this study can be found in online repositories. The names of the repository/repositories and accession number(s) can be found below: BioProject, accession number PRJNA814419.

## Ethics Statement

The studies involving human participants were reviewed and approved by the Ethics committee of the University of Liège Academic Hospital. The patients/participants provided their written informed consent to participate in this study.

## Author Contributions

YA, MG, SR, and GL contributed to the conception and design of the study. EL, MP, and MH contributed to the methodology of the study. VP, SS, and AN performed the statistical analysis. LS and VP performed the bioinformatical analysis. DL and DM performed validation of the result. VP wrote the first draft of the manuscript. VP, SS, LS, YA, SR, and MH wrote sections of the manuscript. All authors contributed to manuscript revision, read, and approved the submitted version.

## Conflict of Interest

YA was a co-founder of PolyOmica and PolyKnomics. GL was the founder and owner of Genos Ltd.,—a private research organization that specializes in high-throughput glycomic analyses and has several patents in this field, and of Genos Glycoscience Ltd.,—a spin-off of Genos Ltd., that commercializes its scientific discoveries. MH and MP were employed of Genos Ltd. MP were also employed by Genos Glycoscience Ltd. The remaining authors declare that the research was conducted in the absence of any commercial or financial relationships that could be construed as a potential conflict of interest.

## Publisher’s Note

All claims expressed in this article are solely those of the authors and do not necessarily represent those of their affiliated organizations, or those of the publisher, the editors and the reviewers. Any product that may be evaluated in this article, or claim that may be made by its manufacturer, is not guaranteed or endorsed by the publisher.

## Footnotes

1
https://genos-glyco.com
