# Supplementary material for: Association Between Human Gut Microbiome and N-Glycan Composition of Total Plasma Proteome
Source: Front Microbiol. 2022 Apr 29;13:811922. doi: 10.3389/fmicb.2022.811922 (PMC9100934; doi:10.3389/fmicb.2022.811922)
Supplement: Supplementary file 2 [file Data_Sheet_2.docx]

Supplementary figures


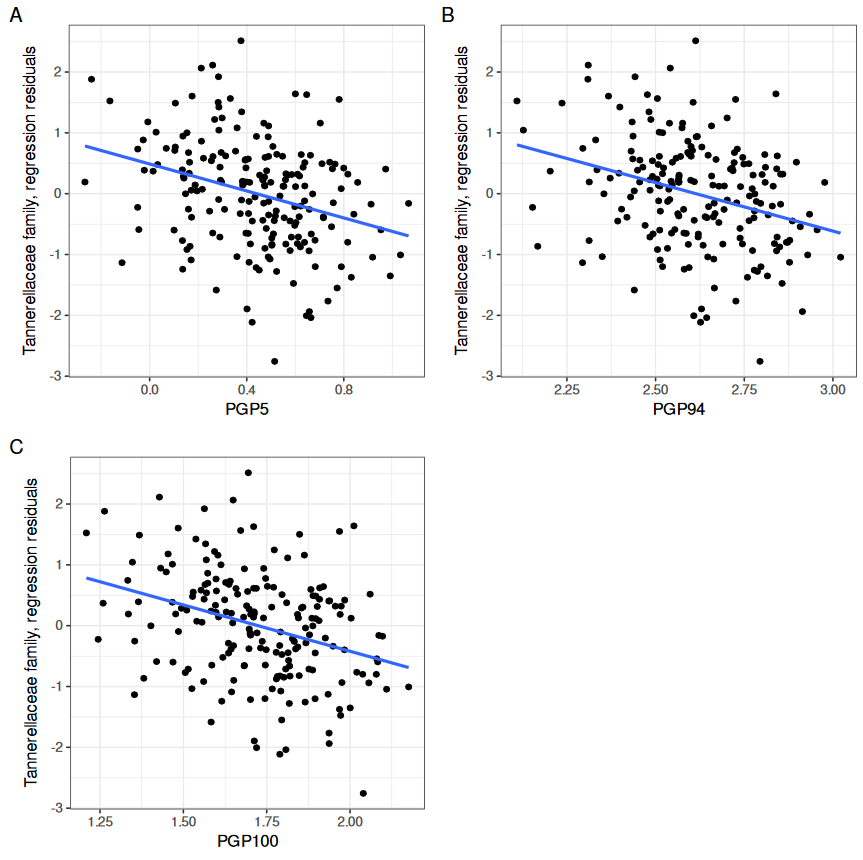


Supplementary figure 1 - univariate associations between microbial families and glycan traits. On the plot, dots represent samples, a regression line shown in black. Panel A - association between the abundance of Tannerellaceae family and the level of FA2[3]G1 in total plasma glycans; panel B - association between the abundance of Tannerellaceae family and the percentage of neutral glycan structures in total plasma glycome; panel C - association between the abundance of Tannerellaceae family and the percentage of monogalactosylated structures in total plasma glycome.


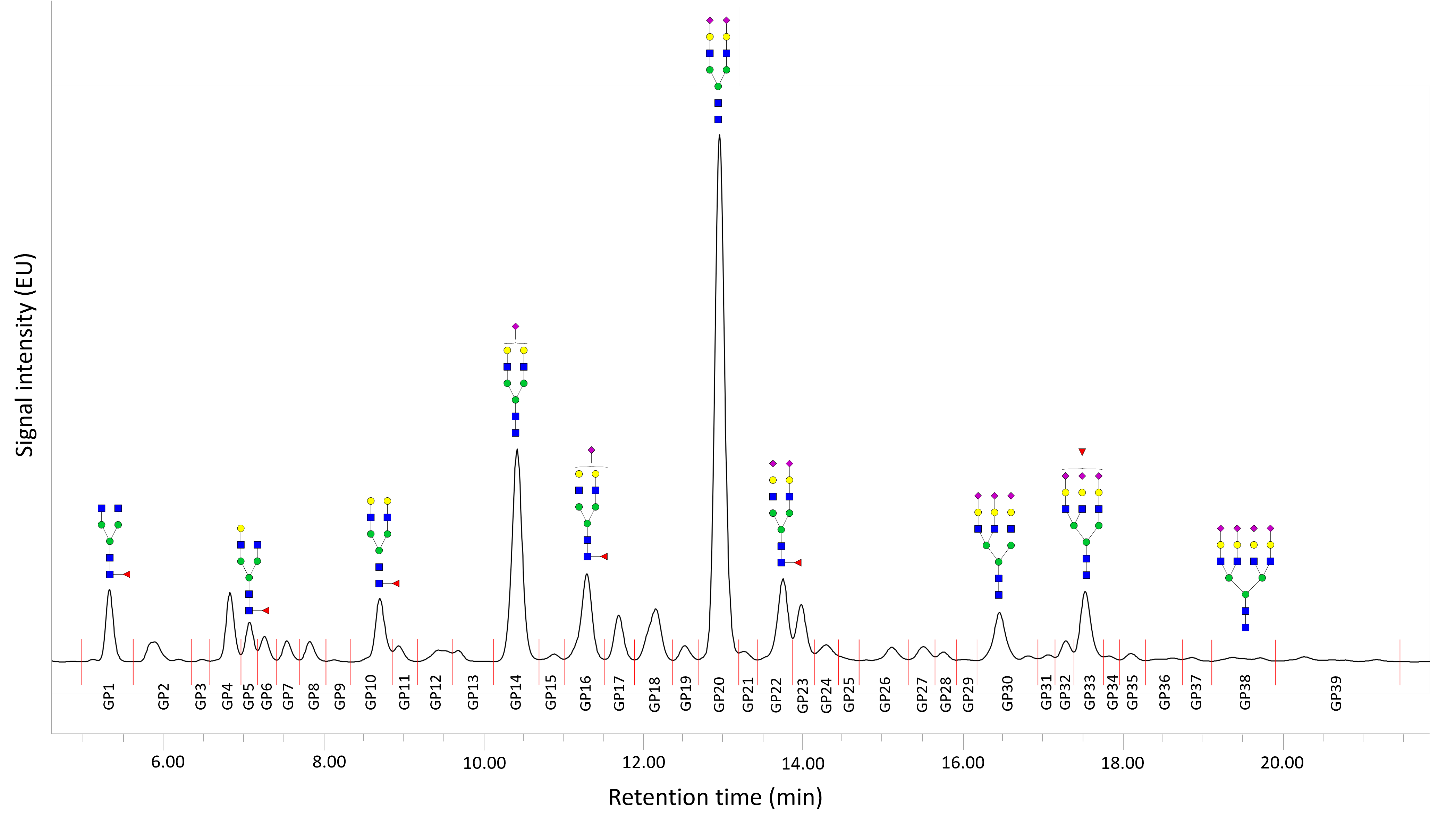


Supplementary figure 2 – representative chromatogram of total human plasma N-glycans
